# *Congou* tea drinking and oesophageal cancer in South China

**DOI:** 10.1038/sj.bjc.6600054

**Published:** 2002-02-01

**Authors:** L Ke, P Yu, Z X Zhang, S S Huang, G Huang, X H Ma

**Affiliations:** Preventive Branch, Shantou University Medical College, Shantou 515031, China; Shantou Epidemic Prevention Station, Shantou 515031, China

**Keywords:** *Congou* tea, alcohol, tobacco, oesophageal cancer, case–control study

## Abstract

The study from a large hospital-based case–control for 1248 cases with oesophageal cancer and the same number of controls in South China showed that *Congou*, a grade of Chinese black tea, may protect against cancers of the oesophagus and reduce the risk of a combination of alcohol drinking and smoking (especially smoking), regardless of temperature when drinking.

*British Journal of Cancer* (2002) **86**, 346–347. DOI: 10.1038/sj/bjc/6600054 www.bjcancer.com

© 2002 The Cancer Research Campaign

## 

There is a high risk region for oesophageal cancer in South China, namely Chaoshan area of Guangdong, with the highest mortality rates of 100 per 10^5^ in Nanao, an island and islet county. Aetiological study is infrequent here. *Congou* is a grade of Chinese black tea, which is habitually drunk at a very high temperature by much (over 80%) of the population in the locality. Concerning the association with tea, several epidemiological and laboratory studies have suggested a protective effect of tea (Inoue *et al*, 1998; Gao *et al*, 1999). However, hot tea was strongly suspected as a risk factor for oesophageal cancer in case–control studies (Stefani *et al*, 1990; Kinjo *et al*, 1998). We made a large hospital-based case–control study to analyze whether mortality risks of oesophageal cancer were associated with hot *Congou* after adjusting other risk factors.

Patients, from the two local hospitals and the three university hospital in-patient departments, were included if they had histological diagnosis of oesophageal squamous cell carcinoma during October 1997 to June 2000 and lived in the country for at least 10 years. For each case, one control, who lived also in the country for at least 10 years, matched by age (±3 years), sex, and same hospital but who had no diagnosis of tobacco and/or alcohol-related diseases.

The same questionnaires for cases and controls were completed by eight trained medical students. Information was collected on occupation, socioeconomic status (income and education); on the lifetime habits of drinking *Congou* tea (quantity, duration, and temperature); alcohol drinking (dose, expressed in millilitres of alcohol, duration, and type of alcoholic beverage); and tobacco smoking (quantity, duration, intensity, and cessation periods). Smoking and dietary habits were assessed in the period just before 10 years before admission to the hospital. A food frequency questionnaire was used to assess the consumption of the various food items. The 12 food groups used in the study questionnaire were included, i.e., sowbelly, kipper, pickled vegetable, smoked food, fermented fish sauce, vegetables, fresh fruits, fresh meat, eggs, cereals, fats, and dairy products. The case–control had been described elsewhere.

An unconditional analysis was carried out because it is generally accepted that in a matched study, this analysis can be performed if appropriate adjustment is made for matching variables which in this study were sex, age, and hospital. Moreover, the results obtained with the conditional analysis were practically identical to those obtained with the unconditional one. Both sexes were combined in statistical analysis.

A total of 1248 cases (median age 58.5 (range 29–82) years, 936 males, 312 female, 85% with habit of *Congou* drinking) and the same amount of controls were included in the study. Unconditional logistic regression analysis with stepwise forward selection (probability for entry 0.05; probability for removal 0.10) of variables showed that those with risk effect were older age, male sex, rural inhabitant, farmer or fisher occupation, lower income, consumption of tobacco, alcohol, fermented fish sauce, pickled vegetable, and sowbelly. Those with protective effect were fresh fruit, cooked vegetable (but lack of dose–response relationship), and *Congou* drinking. An analysis for *Congou* or hot *Congou* drinking after adjustment for age, sex, region, occupation, income, alcohol, tobacco, fish sauce, pickled vegetable, sowbelly, fruit, and vegetable were carried out. Table 1

**Table 1:**
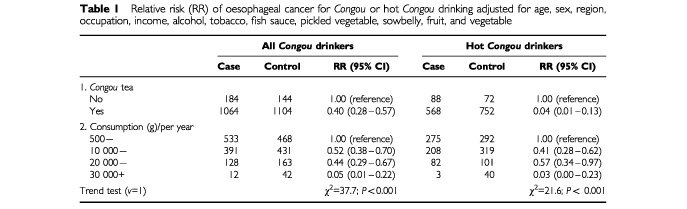
Relative risk (RR) of oesophageal cancer for *Congou* or hot *Congou* drinking adjusted for age, sex, region, occupation, income, alcohol, tobacco, fish sauce, pickled vegetable, sowbelly, fruit, and vegetable

showed a clear protected effect and a significant dose–response relationship with both the consumption of *Congou* and hot *Congou*.

For further analyses, cigarette smoking was divided into non-smoking and current smoking (one cigarette per day or more) after the ex-smokers were excluded. Alcohol drinking groups were divided into either daily or not-daily (three times per week or less) types. In Table 2

**Table 2:**
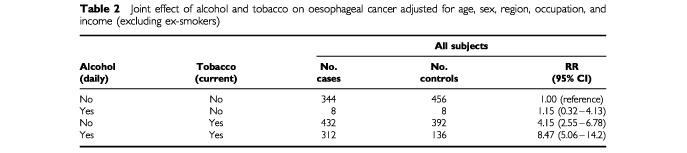
Joint effect of alcohol and tobacco on oesophageal cancer adjusted for age, sex, region, occupation, and income (excluding ex-smokers)

, the RR for daily alcohol drinking and current smoking was 8.47 with 95% CI of 5.06–14.2, relative to men and women exposed to neither habit. Thus, the joint effect of alcohol and tobacco was more than additive (e.g., 8.47>1+ (1.15−1)+(4.15−1)). These results were not significantly affected by further adjustment of the remaining factors.

Thus, the joint effect of alcohol drinking and smoking was a synergy observed in three groups, i.e., non-*Congou*, *Congou*, or hot *Congou* drinking (Table 3

**Table 3:**
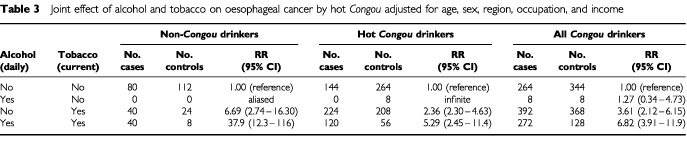
Joint effect of alcohol and tobacco on oesophageal cancer by hot *Congou* adjusted for age, sex, region, occupation, and income

). However, the rate ratios from non-*Congou* drinkers were higher than those from all *Congou* or hot *Congou* drinkers in the same combination of alcohol drinking and smoking, when the subjects with not-daily alcohol drinking and non-smoking were used for the reference. The rate ratios from the subjects with daily alcohol drinking and current smoking were 37.9 (95% CI: 12.3–116), 6.82 (95% CI: 3.91–11.9), and 5.29 (95% CI: 2.45–11.4) for non-*Congou*, *Congou*, or hot *Congou* drinkers, respectively (Table 3). The risk of current smoking among not-daily alcohol drinking subjects seemed to decrease in both *Congou* and hot *Congou* groups, compared with corresponding figure in Table 2.

These results of the present study provide epidemiological support for the hypothesis that tea, regardless of its temperature when drinking, may protect against cancers of the oesophagus, although results from studies that had shown an effect of drinking scalding hot liquids may indicate that the protective effect of tea could be reduced or eliminated (Gao *et al*, 1994).

The results from epidemiology are not consistent, but laboratory studies have indicated that tea has inhibitory effects against tumour formation and growth, for example, the caffeine constituent of black tea has been found to be protective against carcinogenesis for several cancers of a tobacco-specific nitrosamine-NNK in bd-ix rats (Chung *et al*, 1998).
